# Immunogenicity evaluation of inactivated virus and purified proteins of porcine circovirus type 2 in mice

**DOI:** 10.1186/s12917-018-1461-9

**Published:** 2018-04-23

**Authors:** Xiaohui Liu, Ting Ouyang, Teng Ma, Hongsheng Ouyang, Daxin Pang, Linzhu Ren

**Affiliations:** Jilin Provincial Key Laboratory of Animal Embryo Engineering, College of Animal Sciences, Jilin University, Changchun, Jilin, 130062 China

**Keywords:** Porcine circovirus type 2 (PCV2), Inactivated, Subunit, Immunogenicity, Mice, Vaccine

## Abstract

**Background:**

Vaccination is considered as an effective and economical way to against PCV2 infection. However, some of commercial available vaccines are based on inactivated viruses, while the others are based on purified protein of PCV2. In the present study, we aimed to compare the immunogenicity of inactivated virus and purified proteins of porcine circovirus type 2 in mice.

**Results:**

The results showed that positive antiserum titers were significantly increased after second, third and fourth immunization using inactivated PCV2 or purified proteins as coating antigen. Moreover, the inactivated PCV2 induced significantly higher levels of PCV2-specific antibodies than that of PCV2 subunit proteins. After PCV2 wild strain challenged, the average daily gain was comparable with that of mice in the mock group, and the sera from both inactivated PCV2-immunized animals and subunit protein Cap+ORF3 + Rep immunized animals had significantly higher neutralizing antibody titers than that of the PBS group. As expected, the neutralizing antibody in the inactivated PCV2 group was significantly higher than that of the subunit protein group. These results indicated that positive antiserum induced by the inactivated PCV2 had a better reactivity and specificity than that of the positive antiserum induced by the purified proteins.

**Conclusions:**

The results in the present study demonstrated inactivated PCV2 is more effective than PCV2 subunit proteins in stimulating immune response to against PCV2 infection.

## Background

Porcine circovirus 2 (PCV2) is the causative agent of porcine circovirus diseases and porcine circovirus-associated diseases (PCVD/PCVAD), including a number of different syndromes and diseases in pigs, such as post-weaning multi-systemic wasting syndrome (PMWS), porcine respiratory disease complex (PRDC), reproductive failure, granulomatous enteritis, necrotizing lymphadenitis, exudative epidermitis and congenital tremor, which are widely present in every major swine farm [1–3].

To date, the exact mechanisms of PCVD/PCVAD are currently unknown. Although several commercial vaccines based on PCV2a are effective in protecting pigs against a challenge with PCV2a, they cannot protect pigs against the PCV2b genotype that is prevalent worldwide or against other PCV2 genotypes [4, 5]. Therefore, studies focused on an effective vaccine are still urgent and important for PCV2 infection.

Based on current knowledge, many documents were reported either based on inactivated PCV2 or PCV2 subunit proteins [6–9]. However, few studies were reported to compare the efficiency of inactivated PCV2 and PCV2 subunit proteins. Previously, we expressed and purified Rep, ORF3, and different fragments of PCV2 Cap protein [6, 7]. In the present study, we aimed to compare the immunogenicity of inactivated virus and purified proteins of PCV2 in mice. The results of the present study may be useful for production of PCV2 vaccine.

## Methods

### Ethics statement

All animal experiments were prospectively planned and all procedures were carried out under an ethical approval granted by the Animal Care and Ethics Committee of Jilin University (Changchun, China). The Animal Ethics Committee approval number was LSXK2018051.

### Viruses and proteins

The titer of PCV2 strain CC1 [10] on PK-15 cell was 10^9^ TCID_50_/mL. PCV2 strain CC1 was inactivated by 0.4% of formaldehyde (*v*/v) for 72 h at 37 °C.

PCV2 Cap, Rep and ORF3 proteins were prepared and purified according to the protocols described previously [6, 7]. Briefly, the ΔCap17–233 with truncated version of the Cap gene lacking the 5′-end 48 nt encoding the N-terminal 16 amino acid residues was amplified and subcloned into the expression vector pET28b (+) (Novagene, Germany) to generate recombinant expression plasmids pET28b-ΔCap17–233. The ORF3 and Rep genes were amplified and subcloned into the expression vector pET-28c (+) (Novagene, Germany) to generate recombinant expression plasmids, pET-28c-ORF3 and pET-28c-Rep, respectively. The plasmids were transformed into *E. coli* BL21 (DE3) competent cells and expressed with IPTG (1.0 mmol/L) induction. Proteins were purified using HisPur™ Ni-NTA Resin (Thermo scientific) according to the manufacturer’s instruction and stored at 4 °C for use within one week or at − 80 °C for use after a longer time.

### Animal immunization

Specific pathogen-free (SPF) BALB/c mice were purchased from the Laboratory Animal Center, Norman Bethune Health Science Center of Jilin University (Changchun, China). Mice were acclimatized at an environment with a temperature of 24–26 and humidity of 60% for 7 days before experiment.

To compare the immunogenicity of inactivated virus and purified proteins, thirty female BABL/C mice, 5 weeks old, were randomly divided into six groups (5 mice per group), including inactivated virus immunized group (7 log10 TCID_50_/mL, A), Cap+ORF3 immunized group (mol/mol = 1:1, B), Cap+Rep immunized group (mol/mol = 1:1, C), ORF3 + Rep immunized group (mol/mol = 1:1, D), Cap+ORF3 + Rep immunized group (mol/mol = 1:1:1, E), and PBS group (control group), and housed in separate isolation rooms. Preimmune serum was collected prior to immunization. The inactivated virus (100 μL) or purified proteins (100 μg) were emulsified as a 1:1 (*v*/v) mixture with a complete Freund’s adjuvant (Sigma). Mice were immunized subcutaneously with 200 μL of the purified proteins or the inactivated virus. Then, the mice were injected subcutaneously with 200 μL of the proteins or the inactivated virus mixed with incomplete Freund’s adjuvant (Sigma) two week, four weeks and six weeks later, respectively. One week after the fourth immunization, the mice were euthanized by cervical dislocation and the sera were harvested for antibody analyses. The sera were collected and stored in aliquots at − 20 °C.

To further demonstrate whether the inactivated virus and purified proteins induce a sufficiently protective immune response against PCV2, thirty female BABL/C mice, 5 weeks old, were randomly divided into three groups (10 mice per group), including inactivated virus immunized group (9 log10 TCID_50_/mL, A), Cap+ORF3 + Rep immunized group (mol/mol = 1:1:1, B), and negative control group (PBS), and housed in separate isolation rooms. Preimmune serum was collected prior to immunization. The inactivated virus (100 μL) or purified proteins (100 μg, Cap:Rep:ORF3 = 34:43:23) were emulsified as a 1:1 (*v*/v) mixture with a complete Freund’s adjuvant (Sigma). Mice were immunized subcutaneously with 200 μL of the purified proteins or inactivated virus. Then, the mice were injected subcutaneously with 200 μL of the proteins or the inactivated virus mixed with incomplete Freund’s adjuvant (Sigma) two week, four weeks and six weeks later, respectively. 72 h after each immunization, the sera of five mice per group were harvested. Two weeks following fourth immunization, all the mice were infected with 200 μL of PCV2 at 7 log10 TCID_50_/mL or 200 μL of PBS by intramuscular injection, respectively. All mice were observed three times daily for changes in physical appearance and deaths (if any) for up to 14 days post virus exposure. Generally, mice were weighed every other day to determine the average weight change of the group and were observed for clinical signs of distress. Fourteen days post infection, mice were euthanized by cervical dislocation and the sera were collected and stored in aliquots at − 20 °C.

### Assay of mice blood antibody levels

Antibody levels in mice blood was measured by ELISA. Briefly, ELISA plates (Costar) were coated with 100 μL of PCV2 or purified protein, which was diluted to a final concentration of 2 μg/mL in coating buffer (PH = 9.6), at 4 °C overnight and then washed with PBS containing 0.05% Tween 20 (PBST, PH = 7.4) for three times. Unspecific binding of the antibodies was avoided by blocking with 100 μL of 0.5% BSA for 30 min at room temperature. After washing three times with PBST, 100 μL of appropriately diluted serum in PBST containing 0.5% BSA was added and incubated for 30 min at 37 °C. After washing three times with PBST, the bound antibodies were incubated with 100 μL of goat anti-mouse IgG (1:1000). After incubation for 1 h at room temperature and three PBST washes, 100 μL of TMB (Sigma) was added to each well and the mixture was incubated for 15 min at room temperature. The reaction was stopped by adding 100 μL of 1 N sulfuric acid to the mixture, and the optical density at 450 nm was measured (Bio-RAD microplate reader).

### Virus neutralization test (VNT)

Neutralization assay was performed to assess the ability of the serum samples to neutralize the PCV2 according to the protocol described by Jin [11]. Briefly, serum samples were heat inactivated at 56 °C for 30 min, followed by serial dilution in 2-fold increments in Dulbecco’s modified Eagle medium (DMEM) supplemented with 2% of fetal bovine serum (FBS), and mixed 1:1 with 200 TCID_50_ of PCV2 strain CC1 for 2 h at 37 °C. Thereafter, 100 μL of serum-virus mixture was added to 50% confluency of PK-15 cells and incubated for 1 h at 37 °C. After incubation, the serum-virus mixture was removed, and the cells were washed with PBS for three times and further incubated in DMEM supplemented with 2% of FBS at 37 °C and 5% CO_2_. After incubation for 72 h, an indirect fluorescence assay (IFA) was performed as previously described by Yang [12].

### Statistical analysis

Statistical significance was calculated using one-way or two-way analysis of variance (ANOVA), followed by the Bonferroni multiple comparison test. Statistical analysis was performed using GraphPad Prism software, version 5 (GraphPad Software, SanDiego, CA). The results were statistically significant at *p* < 0.05. For each separate set of assays, at least 3 independent experiments were evaluated. The results are expressed as the mean ± standard deviation (SD).

## Results

### Inactivated PCV2 is more effective than four PCV2 subunit proteins in stimulating immune response

To compare immunogenicity, inactivated PCV2 and four different combinations of PCV subunit proteins (different combinations of Cap+ORF3, Cap+Rep, ORF3 + Rep, and Cap+ORF3 + Rep) were used as antigens to raise antiserum, respectively. After four immunizations, the antisera were collected and detected by ELISA using PCV2 as coating antigen. The results showed that the inactivated PCV2 induced significantly higher levels of PCV2-specific antibodies than that of the PCV2 subunit proteins, indicating the inactivated PCV2 was more effective than the PCV2 subunit proteins in stimulating immune response (Table 1). Furthermore, PCV2 subunit protein Cap+ORF3 + Rep showed a better immunogenicity than the other subunit proteins, whereas antibodies were undetectable in the mock groups. Therefore, the inactivated PCV2 and the subunit protein Cap+ORF3 + Rep were selected in the following studies.

**Table 1:** ELISA results (Mean ± SD) of different groups in stimulating immune response

Dilution of Antiserum	Inactivated virus	Cap+ORF3	Cap+Rep	ORF3 + Rep	Cap+ORF3 + Rep	Negative Serum	PBS
1/200	1.1430 ± 0.0463	0.1437 ± 0.0161	0.1033 ± 0.0062	0.0633 ± 0.0045	0.2972 ± 0.0683	0.0649 ± 0.0047	0.0516 ± 0.0004
1/400	1.1476 ± 0.0423	0.1985 ± 0.0557	0.0802 ± 0.0101	0.0554 ± 0.0021	0.1666 ± 0.0431	0.0509 ± 0.0046	0.0558 ± 0.0016
1/800	1.1624 ± 0.0955	0.1712 ± 0.0440	0.0628 ± 0.0037	0.0565 ± 0.0017	0.1132 ± 0.0225	0.0514 ± 0.0012	0.0556 ± 0.0011
1/1600	1.1033 ± 0.0981	0.1146 ± 0.0231	0.0649 ± 0.0054	0.0565 ± 0.0023	0.0550 ± 0.0012	0.0504 ± 0.0016	0.0575 ± 0.0023
1/3200	1.0049 ± 0.1002	0.0802 ± 0.0104	0.0561 ± 0.0016	0.0511 ± 0.0010	0.0512 ± 0.0016	0.0517 ± 0.0009	0.0558 ± 0.0015
1/6400	0.8501 ± 0.0854	0.0634 ± 0.0042	0.0536 ± 0.0011	0.0524 ± 0.0007	0.0541 ± 0.0012	0.0532 ± 0.0014	0.0536 ± 0.0011
1/12800	0.7245 ± 0.0916	0.0567 ± 0.0017	0.0531 ± 0.0007	0.0543 ± 0.0005	0.0557 ± 0.0003	0.0526 ± 0.0006	0.0562 ± 0.0012

Note:Detection limits were underlined. ^*^Positive, Sample/negative serum ≥2

**Fig. 1:**
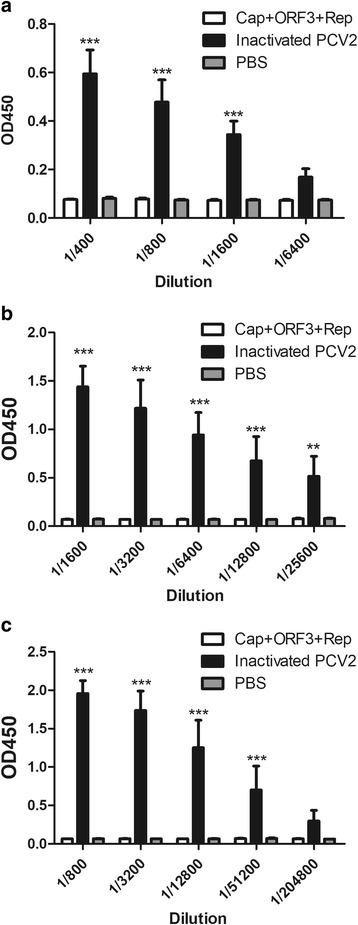
**Serum antibody level after different immunizations in mice.** Inactivated PCV2 and subunit protein Cap+ORF3 + Rep were used as antigens to raise antiserum, respectively. After each immunization, the antisera were collected as described in the Materials and methods section. ELISA was performed using PCV2 as coating antigen. (**a**) Second immune; (**b**) Third immune; (**c**) Fourth immune

**Fig. 2:**
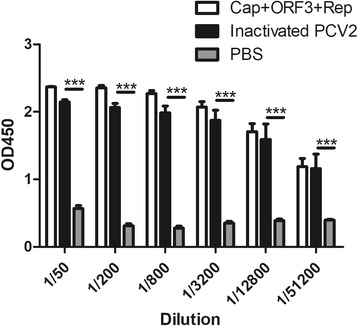
**Serum antibody level after fourth immunization using purified proteins (Cap + Rep, 1:1) as antigen.** Inactivated PCV2 and subunit protein Cap+ORF3 + Rep were used as antigens to raise antiserum, respectively. After fourth immunization, the antisera were collected and analyzed

To compare serum antibody level after immunization in mice, 200 uL of sera were obtained from 5 mice of each group by needle puncture of tail tip vein 72 h after each immunization, followed by ELISA. The results of ELISA showed that positive antiserum titers were significantly increased after second, third and fourth immunization using inactivated PCV2 (Fig. 1) or purified proteins (Fig. 2) as coating antigen. Furthermore, the antiserum developed from the inactivated PCV2 was significantly higher than that of the purified proteins group when inactivated PCV2 was used as coating antigen (Fig. 1). Moreover, when purified proteins Cap+Rep (1:1) was used as coating antigen, the antisera developed from both inactivated PCV2 and purified proteins were significantly higher than that of the control (PBS) group (Fig. 2). These results indicated that positive antiserum induced by the inactivated PCV2 had a better reactivity and specificity than that of the purified proteins.

### Inactivated PCV2 induced more effective immunity than purified subunit proteins against PCV2

**Fig. 3:**
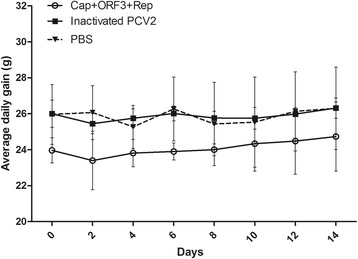
**Average daily gain.** Thirty female BABL/C mice, 5 weeks old, were randomly divided into three groups and immunized subcutaneously with 200 μL of the purified proteins or inactivated virus. Two weeks following fourth immunization, all the mice were infected with 200 μL of PCV2 at 7 log10 TCID_50_/mL or 100 μL of PBS by intramuscular injection, respectively. All mice were observed three times daily for changes in physical appearance and deaths (if any) for up to 14 days post virus exposure. Generally, mice were weighed every other day to determine the average weight gain of the group

To elucidate whether the inactivated PCV2 and purified protein were effective in protecting mice against PCV2, the mice were infected with 200 μL of PCV2 at 7 log10 TCID_50_/mL by intramuscular injection 14 days after the fourth immunization. The results showed that average daily gain had no significant difference between the groups as well as in each group, and the weight gain was not altered by immunization in mice (Fig. 3).

**Fig. 4:**
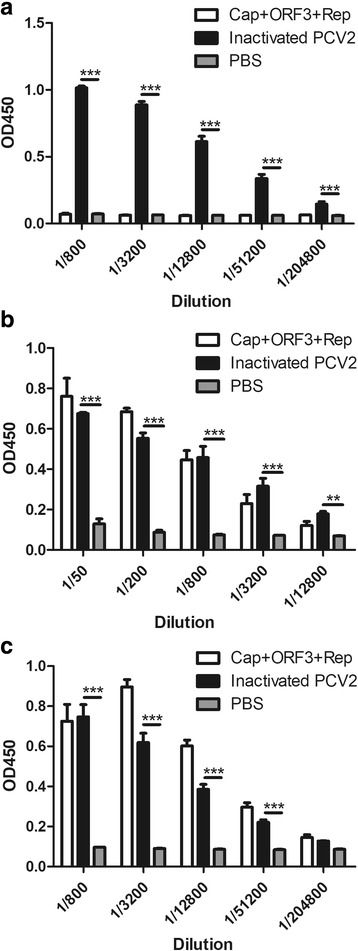
**ELISA results of different groups used as coating antigen. a** Inactivated PCV2 as coating antigen; **b** Cap protein as coating antigen; **c** Rep protein as coating antigen

The antisera from each group were collected and detected by ELISA using inactivated PCV2, Cap protein and Rep protein as coating antigen, respectively. The results demonstrated that serum antibody developed from the inactivated PCV2 showed high levels using inactivated PCV2, Cap protein or Rep protein as coating antigen, respectively (Fig. 4). Furthermore, serum antibody developed from the subunit protein Cap+ORF3 + Rep showed high levels using Cap protein or Rep protein as coating antigen (Figs. 4b and c).

**Fig. 5:**
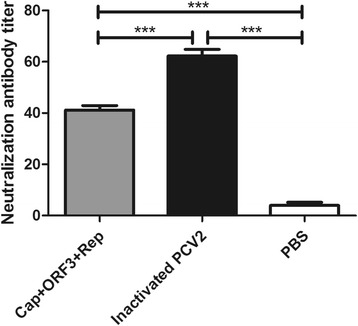
**Neutralizing assay of the immunized sera.** Serum samples were heat inactivated at 56 °C for 30 min, followed by serial dilution in 2-fold increments in Dulbecco’s modified Eagle medium (DMEM) supplemented with 2% of fetal bovine serum (FBS), and mixed 1:1 with 100 TCID_50_ of PCV2 strain CC1 for 2 h at 37 °C. Thereafter, 100 μL of serum-virus mixture was added to 50% confluency of PK-15 cells and incubated for 1 h at 37 °C. After incubation, the serum-virus mixture was removed, and the cells were washed with PBS for three times and further incubated in DMEM supplemented with 2% of FBS at 37 °C and 5% CO_2_. After incubation for 72 h, an indirect fluorescence assay (IFA) was performed to assess the neutralizing activity of the immunized sera. The results represent the means ± S.D. of three replicates of each group. *P*-values were calculated using two-way analysis of variance (ANOVA), followed by the Bonferroni multiple comparison test. *p < 0.05, **p < 0.01, ***p < 0.001

To assess the ability of the serum samples to neutralize the PCV2, neutralization assay was performed and evaluated using IFA. The results showed that the sera from both inactivated PCV2-immunized animals and subunit protein Cap+ORF3 + Rep immunized animals had significantly higher neutralizing antibody titers than that of PBS group (Fig. 5). These results indicated that the inactivated PCV2 and subunit protein Cap+ORF3 + Rep were effective in inducing PCV2-neutralizing antibody responses in mice. Moreover, the neutralizing antibody in the inactivated PCV2 group was significantly higher than that of the subunit protein group. Therefore, these results indicated that immune effect of the inactivated PCV2 was better than the PCV2 subunit protein Cap+ORF3 + Rep in the present study.

## Discussions

The PCV2 genome has eleven open reading frames (ORFs), ORF1 to ORF11 [3]. ORF1 of PCV2 is highly conserved and encodes viral DNA replication-associated proteins, and ORF2 encodes the relatively variable structural protein Cap. Currently, both Rep and Cap are considered as significant immunogenic proteins of PCV2 that play important roles in cell-mediated immunity to constrain PCV2 replication and prevent the progression of PCV2 infection toward PCVD/PCVAD [13–15]. It was reported that PCV2-inoculation induced a cellular immunity against Cap protein as well as Rep protein in pigs [14, 15]. There are eight replication-associated proteins encoded by *ORF1* gene, including two major products (designated Rep and Rep0) and six minor products (designated Rep3a, Rep3b, Rep3c, NS515, NS672, and NS0) [13]. Rep proteins likely contribute to viral pathogenicity in vivo [16]. It was reported that the Rep can not only interact with cellular filament protein and transcriptional regulator c-myc, but also can interact with Cap protein [17], indicating Rep proteins are important in PCV2 infection and pathogenicity. Additionally, Rep protein, especially N-terminus of Rep (RepN), is conserved and immunogenic [18]. However, the N-glycosylation 23–25 aa, 256–258 aa mutation of Rep protein reduced virus replication, but 286–288 aa mutation can enhance virus replication in PK-15 cells [19]. Previously, we found that the reactivity of the antiserum of Rep protein in PCV2 infected cells was significantly better than that of ORF3 [7]. The possible reason was that different native forms encoded by the viral *ORF1* gene in the PCV2-infected cells might enhance the binding affinity of the polyclonal antibody and the replication-associated proteins [7]. These results indicate that the Rep protein is promising for PCV2 antibody and vaccine development [7]. Moreover, many reports showed that ORF3 protein (11.9 kDa) not only involved in PCV2-induced apoptosis by activating caspase-8 and caspase-3 pathways, but also played an important role in viral pathogenesis [3, 20–23]. T lymphocyte responses to PCV2 are primarily directed toward epitopes of Rep and ORF3 proteins [24]. Therefore, we propose that different combinations of PCV2 proteins can induce immunogenicity. In this study, we compared immunogenicity of four different combinations of PCV2 proteins (Cap+ORF3, Cap+Rep, ORF3 + Rep, and Cap+ORF3 + Rep). The results demonstrated that the subunit protein Cap+ORF3 + Rep showed a better immunogenicity than the other PCV2 subunit proteins. Therefore, subunit protein Cap+ORF3 + Rep were selected in the following studies to compare with inactivated PCV2.

Vaccination is considered as an effective and economical way to against PCV2 infection. Some of commercial available vaccines are based on inactive viruses, while the others are based on purified protein of PCV2. Although subunit vaccines are safe, however, their immunogenicity is low [25]. Therefore, it is difficult to choose an economical vaccine. To elucidate whether the inactivated PCV2 and purified protein were effective in protecting mice against PCV2, the mice were infected with PCV2 by intramuscular injection 14 days after the fourth immunization. As expected, the average daily gain was comparable with that of mice in the mock group, and the inactivated PCV2 and the purified protein Cap+ORF3 + Rep can significantly induced PCV2-neutralizing antibody in serum. However, serum antibody developed from the inactivated PCV2 showed higher levels using inactivated PCV2, Cap protein or Rep protein as coating antigen, respectively. Furthermore, the neutralizing antibody in the inactivated PCV2 group was significantly higher than that of the subunit protein group. These results demonstrated that the inactivated virus can induce superior immune responses than that of subunit viral proteins, which were consistent with the results reported by the other groups [26]. The results of the present study indicated that inactivated PCV2 is more effective than purified proteins against PCV2.

Adjuvant is crucial for efficacy of vaccines. In this study, the most commonly used adjuvant, Freund’s adjuvant, was used. The results showed that high levels of specific antibodies were induced in mice, indicating both antigens and adjuvant were effective. There are different classes of adjuvants that can push immune response in different directions, including inorganic compounds, mineral oil, bacterial products, and cytokines, etc. [27, 28]. However, each adjuvant has its advantages and disadvantages. It was reported that GM-CSF as an adjuvant with PCV2 subunit vaccines markedly increases specific humoral immune response [25]. Previously, we found that immunological reactions were significantly increased in HMGCR-inhibited cells and mice [29, 30], and IL-2 can enhance PCV2 infection in vitro *and* in vivo [12, 31]. Thus, we hypothesis that IL-2 used as an adjuvant, combined with downregulated HMGCR, can induce stronger immune response in vivo. Further studies are in progress to test this hypothesis.

## Conclusions

In conclusion, the positive antiserum of inactivated PCV2 had a better reactivity and specificity compared with that of the purified proteins. The inactivated PCV2 can induce better immune response to protect mice against PCV2 infection than that of the PCV2 subunit proteins used in this study. The results in the present study may be useful for production of PCV2 vaccine.

## Abbreviations

ORF: Open reading frame
PCV2: Porcine circovirus 2
PCVD/PCVAD: Porcine circovirus diseases and porcine circovirus-associated diseases
PMWS: Post-weaning multi-systemic wasting syndrome
PPV: Porcine parvovirus
PRDC: Porcine respiratory disease complex
PRRSV: Porcine reproductive and respiratory syndrome virus
SIV: Swine influenza virus
SPF: Specific pathogen-free
TCID: Tissue culture infective dose.
